# Knowledge and utilization of information communication technology (ICT) among health science students at the University of Gondar, North Western Ethiopia

**DOI:** 10.1186/1472-6947-13-31

**Published:** 2013-03-03

**Authors:** Solomon Assefa Woreta, Yigzaw Kebede, Desalegn Tegabu Zegeye

**Affiliations:** 1 Institute of Public Health, Department of Health Informatics, University of Gondar, Gondar, Ethiopia; 2 Institute of Public Health, Department of Epidemiology and Biostatistics, University of Gondar, Gondar, Ethiopia

## Abstract

**Background:**

Despite the relatively huge ICT investment and policy deployment in higher institutions in Ethiopia, there is still scant information about the success of implementation of the Information Communication Technology (ICT) in the higher education. This study, therefore, was carried out with an aim to assess knowledge and utilization of Information Communication Technology (ICT) among medicine and health science students and its associated factors in Gondar College of Medicine and Health sciences, University of Gondar.

**Methods:**

A cross-sectional study was conducted at the College of Medicine and Health Sciences, University of Gondar, Ethiopia. Data regarding socio-demographic characteristics of the students, level of knowledge and utilization of ICT were collected by means of a self-administered questionnaire. Data was analyzed using SPSS version 13.

**Results:**

A total of 1096 students responded giving a response rate of 97.8%. The mean age of the study participants was 20.3 (±1. 3) years. Females constitute only 26% of the respondents. The majority (79%) were fulltime students. Only half of the respondents (51%) had ICT knowledge and only 46% students utilized ICT while 47% of the respondents never used electronic communication (e.g. email or chat room) and 39% of the respondents never used Microsoft office (e.g. word ® or WordPerfect ®). ICT knowledge [AOR = 2.5, 95% CI: 1.7-3.5], family educational background [AOR = 4.36, 95% CI: 2.16-8.80], and perceived quality of training [AOR = 1.9, 95% CI: 1.3-2.8] showed strong and positive associations with ICT utilization. Students from urban areas were more likely to utilize ICT compared with those from rural areas [AOR = 2.7, 95% CI: 2.097, 3.497], and information technology training was found to be positively associated with ICT utilization [AOR = 2. 07, 95% CI: 1.18, 3.62].

**Conclusions:**

The result showed that students’ knowledge was inadequate and utilization of ICT was poor. Therefore, the university should sustain professional development to improve teaching, to raise student performance and equip the college with student centered ICT computer labs to increase students’ ICT utilization.

## Background

The use of ICT to enhance or support learning and teaching in education has become increasingly important in tertiary education [1]. Hence, ICT skills are currently of great interest to governments, businesses and individuals. Information Communication Technology (ICT) has become a powerful tool in the fight against world poverty, providing developing countries with an unprecedented opportunity to meet vital development goals, such as poverty reduction, basic health care, and education, far more effectively than before [2]. Information communication technology in education is a modern, efficient and cost-effective process and has created a need to transform how students from higher institutions learn [3].

Compared with developed countries, the use of ICT in education programs in developing nations is relatively limited. Some of the reasons mentioned for such gaps are because developing countries face shortages of financial resources, limited Internet access, a lack of trained teachers and the lack of proper policies. Nevertheless, there has been growing interest in the use of ICT in educational settings in developing countries. Furthermore, in recent years, several countries have attempted government led initiatives to expand access to ICT in schools. These initiatives have often been associated with a broader educational quality improvement agenda [4].

Like any other developing country, the Ethiopian government has an e-government strategy which covers various sectors such as education, health, agriculture, and public administration [5].

The University of Gondar, as one of the prominent tertiary institutions in Ethiopia, recognizes the broad positive effect of ICT on the quality of learning and is involved in the implementation of various interventions.

The aim of this study is therefore, to assess knowledge and utilization of Information Communication Technology (ICT) among Medicine and Health Science students and its associated factors in College of Medicine and Health Sciences, University of Gondar.

## Methods

The study was carried out at the College of Medicine and Health Sciences, University of Gondar. Currently, the College of Medicine and Health Sciences comprises 4 schools, namely, School of Medicine, School of Public Health, School of Health Sciences and School of Pharmacy. Under these four schools there are 35 departments running 11 undergraduate and 8 postgraduate study programs. In 2011, there were more than 4200 students enrolled in the undergraduate regular, continuing and postgraduate programs in the college. The Department of Health informatics was established in 2005 and since then it has been running the Introduction to Health Informatics course to all undergraduate health Science students. The contents of the course are basic computing, Health information systems and health informatics applications. The department owns 2 computer labs, each with 50 computers and broadband Internet access.

During the study period, the College of Medicine and Health Sciences was teaching more than 4205 graduate and undergraduate students.

Cluster sampling was used to ensure all classes and sexes were included proportionally into the study. Proportional sample numbers of students were taken from all departments and sections. Then the study subjects were selected by systematic random sampling from each department according to alphabetical name lists. Finally, 1119 participants from 3306 fulltime and part time undergraduate students were enrolled in the study.

A structured questionnaire was designed by reviewing the literature. It was pretested on a group of 56 students who did not belong to the selected clusters. The structured questionnaire assessed basic IT skills and knowledge of computer hardware, computer software, computer input devices, computer output devices, basic computer terms and definitions and general understanding of how a computer works. In total 16 questions on ICT knowledge were asked to indicate the level of ICT knowledge, with each statement using a four-point Likert scale. The average score for each question was used to assess knowledge. The average for all 16 ICT knowledge questions was calculated and those with above average score were labeled as knowledgeable while those below were labeled as non-knowledgeable. In this study 11 ICT Utilization questions were also asked to indicate their level of ICT utilization with each statement on a five-point Likert scale. ICT utilization was assessed by using the average score for each of these questions. An average for the 11 ICT utilization questions was calculated and those with above the average score were labeled as utilized and those below were labeled as non-utilized. The study was approved by the Institutional Review Board of the University of Gondar. Oral informed consent was obtained from each participant.

Data analysis was carried out using SPSS (Statistical Package for Social Sciences) version 13. Descriptive analyses were performed for various variables. Bivariate and multivariate logistic regression analysis was conducted. Variables found to have an association (p < 0.2) with the dependent variables, were identified and entered to control the possible confounding effects.

## Results

### Socio-demographic characteristics of students

A total of 1096 students from 11 departments were randomly selected and included in the study, with a response rate of 97.8%. The age range of participants was from 19 to 31 years with a mean (SD) of 20.34 (±1. 38) years. Three quarters of the respondents (74%) were male, 61% were from urban regions and 79% were fulltime students. Three hundred and fifty eight students (33%) were first year students, 365 (33%) in second year and 373 (34%) were in the third year or above.

Socio-demographic characteristics of students are described in Table 1.

**Table 1:** Demographic characteristics of the study population (N = 1096)

**Variable**	**Category**	**N**	**Percent (%)**
Age	15-19	235	21.4
	20-24	818	74.6
	25-29	36	3.3
	30-34	7	0.6
Sex	Male	807	73.6
	Female	289	26.4
Previous residency	Urban	673	61.4
	Rural	423	38.6
Father educational status	Illiterate	180	16.4
	Read and write only	313	28.6
	Primary education	157	14.6
	High school	96	8.8
	Diploma	350	31.9
	Degree and above		
Mother educational status	Illiterate	350	31.9
	Read and write only	290	26.5
	Primary education	119	10.9
	High school	132	12.0
	Diploma	205	18.7
	Degree and above		
Department	Medicine	317	28.9
	Nurse	130	19.3
	Health officer	54	9.3
	Anesthesia	212	3.4
	Physiotherapy	101	1.8
	Midwifery	37	7.8
	Psychiatry	20	2.2
	Optometry	87	3.6
	Laboratory	24	6.8
	Pharmacy	39	11.9
	Environmental and occupation	75	4.9
College attending	Extension	230	21.0
	Regular	866	79.0
Level of education	First year	358	32.7
	Second year	365	33.3
	Third year	198	18.1
	Fourth year	122	11.1
	Fifth year	33	3.0
	Six year	20	1.8
Religion	Orthodox	928	84.7
	Muslim	88	8.0
	Protestant	70	6

### Student knowledge on ICT

It was observed from the study that 51% of the respondents had knowledge of ICT, of which 29% were female and 75% were originally from an urban residence. Of the students that were considered knowledgeable, 42% were full time. Table 2 presents students’ computer and printer access. From the total of 1096 students, 53% reported that they have access to a computer and 91% to a printer. From those who have access to a computer, 6% had a personal computer, 14% had access via an Internet cafe and 28% had access to computers at the university. Table 3 presents responses to questions on computer skills and training. From the overall respondents, 41% were familiar with computers through personal study (self learning) and experience, however 24% were familiar with computers through courses given at the university, and only 8% acquired computer knowledge through special computer training outside the University.

**Table 2:** Response to questionnaire items on computer and printer access amongst a sample of college of medicine and health science students university of Gondar (N = 1096)

**Question**	**Response**	**Male**		**Female**		**Total**	
		**N**	**%**	**N**	**%**	**N**	**%**
Do you have access to a computer?	Yes	413	51	165	57	578	52.7
	No	394	49	124	43	518	47.3
Where do you have access to computers?	At the University	233	29	75	26	308	28.1
	At home	35	4	35	12	70	6.4
	Other place (Internet cafe)	111	14	39	13	150	13.7
	At both home and Internet café	27	3	23	8	50	4.6
How do you describe the access and availability of the computer?	Good / very good	84	10	37	13	121	11
	Adequate	242	30	83	29	325	29.7
	Poor/ very poor	481	60	169	58	650	59.3
Do you have access to a printer	Yes	66	8	34	12	100	9.1
	No	741	92	255	88	996	90.9
How would you describe the access and availability of the printer	Good / very good	133	16	44	15	177	16.1
	Adequate	183	23	78	27	261	23.8
	Poor/ very poor	491	61	167	58	658	60

**Table 3:** Responses to questionnaire items on computer skills and training among CMHS students (N = 1096)

**Variable**		**Utilization**		**COR (95% CI)**
		**Yes**	**No**	
Previous residency	Urban	371	302	2.70(2.09-3.49)*
	Rural	132	291	1
Mother educational status	Illiterate	76	99	1
	Read and write	96	254	1.23(.83-1.82)
	Primary school	125	165	1.41(1.00-1.99)*
	High school	71	48	1.82(1.17-2.85)*
	Higher education	86	46	2.44(1.47-4.07)*
Father educational status	Illiterate	125	80	1
	Read and write	57	123	2.00(1.44-2.78)*
	Primary school	114	199	3.19(2.18-4.67)*
	High school	72	85	3.91(2.53-6.04)*
	Higher education	51	45	4.94(3.22-7.59)*
Computer access in the university	Yes	236	232	1.38(1.07-1.76)*
	No	267	361	1
Computer access at home	Yes	141	55	3.81(2.71-5.34)*
	No	362	538	1
Self rated IT skill	Good / very good	355	122	7.10(4.26-11.81)*
	Adequate	213	320	4.37(3.34-5.72)*
	Poor/ very poor	25	61	1
Ability to use word processor	Competent in most skill	38	32	8.85(5.36-4.60)*
	Competent in basic skill	41	47	7.44(5.61-9.86)*
	Unable beginner	416	513	1
ICT familiarization	Through a course in the university	96	254	1
	Personal study and experience	125	165	2.20(1.59-3.04)*
	Special course	71	48	2.22(1.36-3.63)*
	Course in the university, personal study, ,experience and special study	86	46	4.14(2.61-6.56)*
	Special course, personal study, and experience	125	80	2.12(1.30-3.47)*
	Course in the university and experience			2.94(1.77-4.86)*
Access to computer in the café and other non specific place	Yes	316	262	2.13(1.67-2.72)*
	No	182	331	1
Knowledge	Knowledgeable	164	284	1
	Not knowledgeable	171	567	6.34(4.86-8.26)*
Quality of teaching in computer in the university	Good / very good	355	122	4.02(2.85-5.67)*
	Adequate	213	320	3.90(2.94-5.17)*
	Poor/ very poor	25	61	1
How do you describe the access computer and printer	Good / very good	38	32	2.70(1.80-4.04)*
	Adequate	41	47	1.51(1.16-1.98)*
	Poor/ very poor	416	513	1

Among all respondents, 49% self rated their basic general IT skills as competent in some basic IT skill, 8% as competent in most basic IT skills, 44% rated themselves as less competent or a beginner in general IT skills and half of the respondents described their IT training as poor.

Table 3 presents responses to questions on computer skills and training.

### Student ICT utilization

Table 3 presents responses to questions on computer skills and training. Around a quarter (23%) of the study population had been using computers for more than three years. One quarter of the respondents (29%) reported irregular use of computers. Almost half of the subjects (49%) were competent in some basic skills. About 10% of the students responded that, they use a computer on daily basis, whereas 33% use a computer once a week and nearly 41% the students use a computer once in a month or very few times in a year. About half of the students (47%) have never used any form of electronic communication. From the total study participants only 8% used electronic communication like email and chat almost every day.

The results also showed that among the electronic communication means, email or chat services are the most widely used applications (51%), followed by the Internet search engines to look up academic information (49%) and 38% of students utilize the Internet to look up information about people, things and ideas.

The computer center at the College of Medicine and Health Sciences was visited almost every day by only forty three (4%) of the respondents, about 24% used the computer centre once a month and 20% of the respondents visited the centre once a week. 8% of the students used the centre 2–3 days per week, but 45% of the respondents never used the centre at all.

### Factors associated with the utilization of ICT

Table 4 presents factors associated with the utilization of ICT. Binary logistic regression was used and those whose p values were less than or equal to 0.2 were fitted to multiple logistic regression. The regression analysis showed that those students who came from educated families were almost four times more likely to utilize ICT compared to non educated family [AOR = 4.36, 95% CI: 2.16-8.80]. Those students competent in most general IT skill were almost twice as likely to utilize ICT compared with less competent or beginner general IT skill students, [AOR = 1. 63, 95% CI: 1.13-2.34]. Students competent in word processing were six times more likely to utilize ICT compared to those without this skill [AOR = 5. 83, 95% CI: 3.15- 10.79].

**Table 4:** Factor associated with the utilization of ICT among CMHS students (N = 1096)

**Variable**		**Utilization**		**COR (95% CI)**	**AOR (95% CI)**
		**Yes**	**No**		
Previous residency	Urban	371	302	2.70(2.09-3.49)*	1.03(.687-1.55)
	Rural	132	291	1	1
Maternal educational status	Illiterate	76	99	1	1
	Read and write	96	254	1.23(.83-1.82)	1.59(1.01-2.52)*
	Primary school	125	165	1.41(1.00-1.99)*	2.31(1.19-4.48)*
	High school	71	48	1.82(1.17-2.85)*	4.36(2.16-8.80)*
	Higher education	86	46	2.44(1.47-4.07)*	2.05(1.02-4.13)*
Self rated IT skill	Good / very good	355	122	7.10(4.26-11.81)*	1.63(1.13-2.34)*
	Adequate	213	320	4.37(3.34-5.72)*	1.20(.60-2.40)
	Poor/ very poor	25	61	1	1
Ability to use word processor	Competent in most skill	38	32	8.85(5.36-4.60)*	4.73(3.37-6.64)*
	Competent in basic skill	41	47	7.44(5.61-9.86)*	5.83(3.1510.79)*
	Unable beginner	416	513	1	1
Knowledge	Knowledgeable	164	284	1	1
	Not knowledgeable	171	567	6.34(4.86-8.26)*	2.47(1.72-3.55)*
Perceived quality of teaching in computer in the university	Good / very good	355	122	4.02(2.85-5.67)*	2.49(1.60-3.86)*
	Adequate	213	320	3.90(2.94-5.17)*	1.96(1.36-2.84)*
	Poor/ very poor	25	61	1	1

Students who had ICT knowledge were twice more likely to utilize ICT compared to without this knowledge [AOR = 2.47, 95% CI:1.72-3.55], and students with perceived good quality of IT education were two times more likely to utilize ICT compared to those who perceived their education to be of poor quality [AOR = 1.96, 95% CI: 1.36-2.84].

## Discussion

The present study examined the knowledge and utilization of ICT and its associated factors in CMHS, University of Gondar. The result of this study revealed that half of students had ICT knowledge, which is similar to the study conducted in Medical school of Ahmadu Bello University, Zaria, Nigeria [6] and a bit lower than a study conducted in India (58%) [7]. Despite the introduction of IT into the curriculum of preparatory schools and in the universities, the level of computer literacy among CMHS students is very low. This perhaps may due to poor access to computer among the students or the inadequacy of the IT courses provided in the university [8].

The study also showed utilization dissimilarities among students in urban and rural areas. This is similar to a study in Ghana where students in urban areas have more positive attitudes towards ICT use compared to students in rural areas [9]. The difference in utilization may be explained by the availability of ICT facilities (such as Internet connectivity, electricity, telephone etc.) in the urban areas as compared to the rural areas in most developing countries. In addition, people are more likely to respond positively to something or someone after increased exposure for example to ICT tools in this case.

Among CMHS students half of the respondents (49.5%) describe their IT training as poor and only 18% describe it as good, which is quite different from the study conducted on students ICT knowledge familiarity in a dental institution in India, where nearly half the subjects (48%) reported that the quality of IT training received was adequate [10].

Hence, we recommend the college to review the ICT training run in the college and make necessary adjustments based on these findings. The majority of the students do not have their own computers, and only 47% of the students have access to computers in college. This figure is low and comparable to study carried out in Nigeria [6]. This might be due to financial constraints preventing the students from owning a computer. In addition, the low utilization of computers might be the inadequacy of computer labs and computers in the college where there are only two computer laboratory each with about 50 computers for all the students in the college. If students do not have access to ICT then many of the perceived advantages of using ICT for education will not translate into reality.

This study also has shown that only 46% of respondents could use a computer, which is similar to the study conducted in a Nigerian teaching hospital which showed that among first year clinical and nursing students only 43% [11] could use computers. However it is lower when compared to studies carried out among medical students in Malaysia which showed 61% could use a computer [12]. As discussed earlier, access to computers, previous residency, knowledge about ICT and maternal education contribute to such low levels of utilization [13].

The findings in this study have shown, only half of the students ever used electronic communication (e.g. E-mail or “chat rooms”). This finding corresponds to the study carried out in Coastal South India (51%) [14] but is lower than the studies conducted in Nigeria (76.4%) [11] and Hadramout University, Yemen (76.2%) [15]. The reason for this perhaps is the limited access to computers and cost of Internet services. The results of this study also revealed that half of the students used Internet to look up academic information, which is lower than the study conducted in Lahore, Pakistan 61.0% [16]. There may be a number of reasons for this difference in use of Internet. Problems such as lack of access to Internet, limited training in how to use the Internet, or absence of tasks set by the instructors that require use of online databases such as UpToDate may have all contributed.

Hence, emphasis should be placed on accommodating training in ICT as well as ICT-enabled teaching and learning. ICT should be taught as a subject, and integrated as a pedagogical tool for teaching and learning in other subject areas.

## Conclusion

Students in the College of Medicine and Health Sciences, University of Gondar had inadequate knowledge of ICT and very low ICT utilization. Establishing an undergraduate students’ computer lab, incorporating information-based discipline, and strengthening health informatics training would equip them with the skills they need to utilize ICT. CMHS leaders should be aware of this existing gap, and start to bring fundamental changes in their institutions with regard to ICT. Further research should focus on designing and evaluating computer and IT training for students.

Though, this study is the first of its kind in our college and probably in the nation, it has its own limitations. Firstly the study depends on self-reported data, which might be susceptible to recall bias. Secondly the questions we prepared to assess knowledge and utilization may not be comprehensive enough to assess all the issues correctly.

## Abbreviations

AOR: Adjusted Odd Ratio;CMHS: College of medicine and health sciences;CI: Confidence Interval;ICT: Information communication technology;IT: Information technology

## Competing interests

The authors declare that they have no competing interests.

## Authors’ contributions

SAW conceived, designed and coordinated the study, participated in data acquisition, carried out data collection, analysis and interpretation, drafted the manuscript. YKG and DTZ participated in designing the study, review of the manuscript, and analysis. All the authors have read and approved the final manuscript.

## Pre-publication history

The pre-publication history for this paper can be accessed here:

http://www.biomedcentral.com/1472-6947/13/31/prepub
